# Developing and Evaluating a Capacity-Building Intervention for Healthcare Providers to Improve Communication Skills and Awareness of Hard of Hearing and D/deaf Populations: Protocol for a Participative Action Research-Based Study

**DOI:** 10.3389/fpubh.2021.615474

**Published:** 2021-04-28

**Authors:** Patrick Bodenmann, Pascal Singy, Miriam Kasztura, Madison Graells, Odile Cantero, Kevin Morisod, Mary Malebranche, Pascal Smith, Stéphane Beyeler, Tanya Sebaï, Véronique S. Grazioli

**Affiliations:** ^1^Department of Vulnerabilities and Social Medicine, Center for Primary Care and Public Health, University of Lausanne, Lausanne, Switzerland; ^2^Psychiatric Liaison Service, Lausanne University Hospital, Lausanne, Switzerland; ^3^Department of Medicine, Cumming School of Medicine, University of Calgary, Calgary, AB, Canada; ^4^Schweizerischer Gehörlosenbund-Fédération Suisse des Sourds (SGB-FSS), Lausanne, Switzerland

**Keywords:** D/deaf, hard of hearing, healthcare staff, capacity building intervention, participative action research

## Abstract

**Background:** D/deaf and hard of hearing populations are at higher risk for experiencing physical and mental health problems compared to hearing populations. In addition, they commonly encounter barriers to accessing and benefiting from health services, which largely stem from challenges they face in communicating with healthcare providers. Healthcare providers commonly lack tailored communication skills in caring for D/deaf and hard of hearing populations, which lead to difficulties and dissatisfaction for both staff and D/deaf and hard of hearing communities. This research project aims to develop and evaluate a capacity-building intervention for healthcare providers with the goal of increasing their awareness of D/deaf and hard of hearing individuals' experiences with the healthcare system, their distinct needs, and improving their capacity to communicate effectively with this patient population.

**Methods:** This research project features a participative action research design using qualitative and quantitative methods. Consistent with participative action research, the study will actively involve the target populations, key stakeholders and representative associations. The intervention will be developed and tested through iterative phases. The Integrated Model of Training Evaluation and Effectiveness will guide prospective evaluation of the intervention. The latter will involve qualitative and quantitative assessments in participants before and after the intervention and at 6-months follow-up.

**Discussion:** Results will contribute to research aimed at decreasing barriers to accessing and benefiting from healthcare services for D/deaf and hard of hearing individuals. Findings will be presented to representative associations and political authorities, as well as disseminated at research conferences and in peer-reviewed journals.

## Introduction

Around 466 million people experience some type of hearing loss worldwide [i.e., hearing loss > 40 and 30 dB in the better hearing ear in adults and children, respectively; (1)] and this number may reach 900 million by 2050. This group includes D/deaf and hard of hearing individuals who self-identify themselves as part of the hearing population (lowercase *d*) or as part of the Deaf community (with a capital *D*), a minority community with its own culture. Among these groups, Deaf community communicates with sign language, whereas deaf and hard of hearing individuals communicate with vocal language while using hearing assistive devices or communication tools stemming from the local language. In Switzerland, more than one in 100 individuals (0.1%) are D/deaf, whereas up to 13% of the population are hard of hearing (2, 3).

Epidemiological studies have documented a wide range of health inequities in D/deaf and hard of hearing populations. Findings indicate for instance that they are more likely to engage in risky behaviors than hearing populations [e.g., heavy alcohol use, unsafe sex; (4, 5)]. Furthermore, D/deaf and hard of hearing populations are at higher risk of experiencing mental [e.g., depression; (6–8)], and physical health problems [e.g., chronic diseases; (7, 9–11)] comparing to hearing populations.

Alarmingly, despite their need of services, D/deaf and hard of hearing populations experience significant barriers when accessing healthcare services that negatively impact their access to care and health outcomes (12, 13). The most significant barriers stem from communication challenges commonly experienced by both D/deaf and hard of hearing populations, and healthcare providers during clinical encounters (14–16). Examples of challenges include using complex medical terminology or drug prescription instructions, which are not understood by the patient, potentially leading to preventable adverse events (17). Communication and language difficulties also lead to unsatisfactory patient-provider relationships (18, 19). A recent survey conducted in 21 public and private hospitals and ambulatory care centers in the *Canton of Vaud* (a French-speaking region of Switzerland comprising ~10% of the Swiss population) documented healthcare providers' difficulties in communicating with the target population (20). Similar findings were yielded in another study conducted in the same region that also highlighted dissatisfaction regarding healthcare in the target population (21).

Healthcare providers commonly lack tailored communication skills, knowledge about D/deaf culture and D/deaf and hard of hearing individuals' experience and specific needs (16, 18, 20, 22, 23). This is critical because lack of knowledge has been associated with decreased access to healthcare among individuals with hearing loss (12, 15, 24, 25). Despite its importance, there is currently no formal capacity-building intervention for healthcare providers in the *Canton of Vaud* in Switzerland aimed at raising awareness about the barriers faced by D/deaf and hard of hearing individuals' in the healthcare system and improving communication (21).

Healthcare providers may use existing educational support offering practical advice and guidance on how communicating with D/deaf and hard of hearing patients [e.g., (26)]. However, although helpful for interested professionals, this education support is likely to reach a very limited number of healthcare providers who are typically overworked. Developing and implementing interventions in the healthcare system is critical to reach a broader audience. However, scarce research to date focused on developing and evaluating training to improve healthcare providers' communications skills with these populations. To the best of the authors' knowledge, only a handful of previous studies focused on developing such interventions and they involved students [i.e., medical and pharmacist students; (27, 28)]. These studies aimed to develop a role-reversal exercise in which students were the patients and Deaf volunteers the medical staff. Findings documented a high level of satisfaction among students, with up to 97% reporting that this experience would have an impact on their attitudes and behaviors in the future (27, 28). However, no longitudinal evaluation was conducted and this program was not tailored to healthcare providers already in the field.

Given the lack of research in this area and the negative experience of D/deaf and hard of hearing individuals while receiving healthcare, an important next step for research is to develop and test interventions tailored to healthcare providers. In response, this research project aims to develop and evaluate a capacity building intervention for healthcare providers to improve their communication skills and increase their awareness of D/deaf and hard of hearing individuals' experience and specific communication needs. The research project will feature a participative action research design. Participative research is particularly critical when it comes to addressing health inequities in minority groups (29). Participative-based research aims at conducting research with communities in a collaborative and equitable partnership, allowing power-sharing process, and building on strengths and resources of the communities (30). Using this design will ensure to develop an intervention that will rely on strengths and resources within the involved community and that will be tailored to their specific needs and expectations. Accordingly, both the content and the format of the capacity building intervention will be developed together with the target populations, key stakeholders and representative associations. The resulting capacity building intervention will be evaluated among healthcare providers (e.g., reception staff, physicians, nurses, pharmacists) using a qualitative and quantitative two-fold measure. First, the intervention content and design (i.e., satisfaction and perception of the intervention content, quality and appropriateness) will be evaluated both quantitatively and qualitatively after receiving the intervention. Second, perception of self-efficacy regarding the skills taught during the intervention and organizational payoffs (i.e., frequency of use or intention to use the skills taught during the intervention) will be measured quantitatively before the intervention, directly after and 6 months later to evaluate changes in participants after receiving the intervention.

## Methods and Analysis

### Setting and Study Design

#### Setting

This research project^1^ will take place at the Center for Primary Care and Public Health in Lausanne (Unisanté) in collaboration with several medical institutions involved with the target population in the *Canton of Vaud*, D/deaf and hard of hearing representative associations and D/deaf and hard of hearing individuals. Consistent with participative research, the research team will be multidisciplinary, including staff from the medical, linguistic and psychology sciences, and will include D/deaf, hard of hearing and hearing staff.

#### Action Research Design

**Figure 1:**
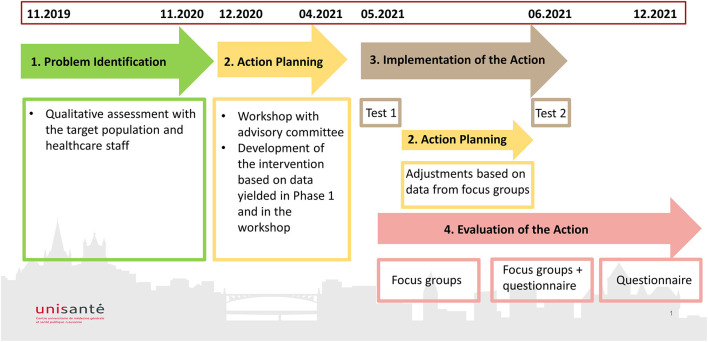
Research project timeline by research action phases.

This project will feature an action research design using qualitative and quantitative methods. Action research is aimed at solving practical concrete problems, enhancing staff knowledge and skills, empowering participants and improving the quality of care (31). The action research design features five cyclical and iterative phases (see Figure 1): (1) *Problem identification* (identify and define the problem); (2) *Action planning* (design the action based on phase 1 data); (3) *Implementation of the action*; (4) *Evaluation of the action*; and (5) *Learning and refinement* (i.e., action refinement and dissemination) (32).

#### Patient and Public Involvement

Action research allows for a bottom-up approach, involving the target population, the stakeholders and the researchers to better understand and address an identified problem through active collaboration (33). Accordingly, the research team will include three D/deaf and hard of hearing research collaborators who will be involved in the intervention development. Together with the hearing research staff, they will develop the content and format of the intervention based on results yielded in Phase 1. Furthermore, the three D/deaf and hard of hearing research staff will be the trainers during the intervention. Finally, they will be involved as co-authors in findings' dissemination related to intervention development and evaluation (i.e., presentations, critical review of manuscripts and co-authorship). Participative research will also be operationalized through establishing an advisory committee including D/deaf and hard of hearing individuals, association representatives, involved stakeholders and experts. This committee will provide ongoing feedback and advice to the research team and will also participate in the intervention development.

As shown in this Figure 1, the project started in November 2019 and is scheduled to be completed in 2 years and 2 months over five phases of research action.

### Study Procedures in Phase 1: Problem Identification

#### Institutional Partnerships

The research team previously created partnerships with 5 medical institutions involved with the target population in the *Canton of Vaud* including clinical units from the home institution; geriatric acute services and geriatric rehabilitation unit at Lausanne University Hospital, the local nursing home association (HévivA) and the local home support association (AVASAD). Heads of partner institutions will provide support to the research team to promote the research study within the institutions.

#### Advisory Committee

The research team previously formed an advisory committee including two D/deaf and one hard of hearing individuals; two healthcare providers with experience with the target population; a representative of administrative healthcare staff; representatives from D/deaf and hard of hearing-focused associations; and one interpreter in French sign language. The committee will be involved in the development of the intervention and will provide ongoing feedback through regular meetings.

#### Phase 1 Data Collection

The research team will conduct qualitative assessments with healthcare staff and the target population to clarify the barriers, needs and gather advice on the intervention content being developed. This phase will aim to understand and describe the issue and needs from the perspective of the target population.

#### Healthcare Staff Participants

Healthcare providers and administrative staff from the partner institutions will be invited to participate in one-on-one semi-structured interviews. Inclusion criteria include being 18 years or older and having cared for or assisted at least one D/deaf or a hard of hearing individual in a healthcare work environment. A group characteristics sampling per quotas by functions (i.e., physicians, nurses, other) was selected as purposeful sampling. We expect that between 5 to 10 interviews per type of healthcare staff will be necessary to achieve saturation, leading to around 15–30 interviews.

#### D/deaf and Hard of Hearing Individuals

Inclusion criteria include being 18 years or older and being D/deaf or hard of hearing. A group characteristics sampling per quotas by hearing loss (i.e., D/deaf vs. hard of hearing) was selected as purposeful sampling. We added a group characteristics sampling per quotas by age (i.e., 18–64 vs. 65 and older) for hard of hearing individuals to account for age-related hard of hearing (i.e., around 65) vs. more precocious forms of hard of hearing. We expect that between 5 to 10 interviews per quota will be necessary to achieve saturation, leading to 15–30 interviews.

Recruitment will be conducted with the support of D/deaf and hard of hearing-related associations and within the participating nursing home. In addition, a short video promoting study participation in sign language will be posted on the websites of D/deaf associations. When first getting in touch with interested participants, the research team will ask them to indicate their preferred mode of communication for the one-on-one semi-structured interviews. When necessary, the research staff will conduct semi-structured interviews with French sign language interpreters.

#### Phase 1 Qualitative Analysis

Semi-structured interviews will be audio recorded and transcribed *verbatim*. Personal identifiable information will be removed from transcripts prior to data coding. We will use conventional content analysis to analyze data (34). The research team will code the data using a constant comparative process (35). Initial coding will be conducted separately using line-by-line technique, whereby coders narrate the actions occurring in the data. Following independently initial coding, we will create a codebook in consensus meetings, pooling incident-by-incident codes and removing collapsing idiosyncratic or redundant codes. Next, we will use the codebook to independently double-code 10% of the sessions. After reaching adequate intercoder consistency (80%), a single coder will code the remaining sessions. Finally, feedback from the advisory committee will serve as a means of assessing fit and resonance of findings.

This process will be conducted independently with data yielded among healthcare and administrative staff and with data collected in the target population. Qualitative analysis will be conducted using Atlas.ti software.

### Study Procedures in Phase 2: Action Planning

This phase will start with workshop involving the advisory committee aimed at defining a concept for the capacity building intervention and creating potential solutions. The research team will then rapidly develop a prototype intervention, based on ideas generating through the workshop and qualitative findings from Phase 1. The research team will organize the first capacity building intervention that will be tested in the next phase (Phase 3) through partner institutions.

### Study Procedures in Phase 3: Action Implementation

A first round of the capacity building intervention will take place in the partner institutions. We expect around 2 participants from each partner institution to participate in the first round of testing. After the intervention, participants will complete a short questionnaire and participate in a focus group to evaluate intervention's content, quality and appropriateness and assess their overall satisfaction (see Table 1 and Phase 4 for more details regarding the evaluation).

**Table 1:** Summary of measures by action research phase.

**Components**	**Tool**	**Participants**	**Timing of assessment**
**Phase 1: Problem Identification**			
Needs analysis	Qualitative assessment (one-on-one semi-structured interviews)	Healthcare staff (*n* ~ 15) D/deaf and hard of hearing individuals (*n* ~ 15)	Beginning of Phase 1
Qualitative assessment (semi-structured interviews)	Healthcare staff (*n* ~ 15) D/deaf and hard of hearing individuals (*n* ~ 15)	Beginning of Phase 1	
**Phase 2: Action Planning**			
No measure			
**Phases 3 and 4: Action implementation and Evaluation**			
**Intervention Testing: Round 1**			
**Participants' reactions**: perception of intervention's content, quality and appropriateness, overall satisfaction	Adapted IMTEE questionnaire^*a*^ Qualitative assessment (focus group)	Healthcare staff receiving intervention 1 (*n* ~ 10)	After intervention 1
**Intervention Testing: Round 2**			
**Participants' reactions**: perception of intervention's content, quality and appropriateness, overall satisfaction	Adapted IMTEE questionnaire^a^ Qualitative assessment (focus group)	Healthcare staff receiving intervention 2 (*n* ~ 50)	T1^d^ T1
**Changes in participants**: perception of self-efficacy, attitudes toward skills taught during the intervention	Adapted IMTEE questionnaire^b^ Qualitative assessment (focus group)		T0, T1, T2^d^ T1
**Organizational payoffs**: frequency of use or intention to use skills taught in the intervention	Adapted IMTEE questionnaire^c^ Qualitative assessment (focus group)		T0, T1, T2^d^ T1

a *Participants will be asked to rate their satisfaction regarding the intervention and to evaluate its content, quality, and appropriateness using a 5-point Likert scale, ranging from 1 (not satisfied at all/improvement needed) to 5 (completely satisfied/very good)*.

b *Participants will be asked to indicate the extent to which they agree with items using a 5-point Likert-scale ranging from 1 (strongly disagree) to 6 (strongly agree)*.

c *To evaluate intentions to use the newly acquired skills, we will ask participants to indicate the extent to which they agree or disagree with statements using a 6-point Likert scale ranging from 1 (strongly disagree) to 6 (strongly agree). Regarding frequency of use, participants will be asked to indicate how often over the past 6 months they applied specific strategies, using a 5-point Likert scale ranging from 1 (never) to 5 (most of the time)*.

d *T0 = before intervention 2; T1 = just after intervention 2; T2 = 6 months after intervention 2*.

A second improved version of the intervention will then be developed based on the findings yielded in the first round of testing and through discussions with the advisory committee. A second round of the intervention will be organized to test it (involving new participants); for this second round, we expect including between 5 and 15 participants by partner institution (*n* ~ 50). The same methods will be applied to evaluate the improved version of the intervention.

### Study Procedures in Phase 4: Action Evaluation

The theoretical framework that will guide the intervention evaluation is the *Integrated Model of Training Evaluation and Effectiveness* (IMTEE) (36). According to the IMTEE, the needs' analysis' results (i.e., data gathered during Phase 1) inform the content and design of the intervention, which enhances changes in participants and in organizational payoffs. Accordingly, the intervention evaluation will examine *intervention content and design* (i.e., satisfaction and perception of the intervention content, quality and appropriateness); *changes in participants* (i.e., perception of self-efficacy, attitudes toward skills taught during the intervention); *organizational payoffs* (i.e., frequency of use or intention to use skills taught in the intervention).

The questionnaire that will be used to evaluate the intervention will be developed to fit intervention specific content and adapted from past research using the IMTEE framework (37, 38). Once developed, it will be pre-tested in healthcare providers (*n* = 10) to ensure items' understanding. Table 1 presents measures assessment by phase.

#### Data Collection After the First Round of Capacity Building Intervention

Participants will be asked to rate their satisfaction regarding the intervention and to evaluate its content, quality and appropriateness (37). They will also be invited to participate in a focus group shortly after the intervention.

#### Quantitative and Qualitative Analysis (First Round of Intervention)

Descriptive statistics will be used to analyze quantitative data assessed with the questionnaire, whereas qualitative data yielded from focus groups will be analyzed using the same methods described in Phase 1.

The second version of the intervention will be refined based on results with the support and guidance of the advisory committee, which will lead to the creation of the second and final version of the intervention.

#### Data Collection After the Second Round of Intervention

For the testing of the second intervention, quantitative data will be collected at pre-intervention (T0), post-intervention (T1, just after the intervention) and at 6-month post-intervention (T2), whereas a qualitative assessment will be conducted at T1 (see Table 1). A questionnaire will be used to assess the following dimensions:

##### Intervention Content and Design

We will adapt and use the questionnaire from the first round of testing to evaluate reactions of participants regarding the intervention at T1 (36).

##### Changes in Participants

We will use 3–5 items to measure perceived self-efficacy to apply the skills taught during the intervention, 3–5 items to evaluate perceived acquisition of knowledge and 3–5 items to assess attitudes toward the intervention content (37, 38) at T0, T1 and at T2.

##### Organizational Payoffs

We will use 6–10 items to assess the intention to use and use frequency of the newly acquired skills use over the past 6 months (37, 38) at T0, T1, and T2.

These dimensions will also be evaluated qualitatively through focus group at T1.

#### Quantitative and Qualitative Analysis (Second Round of Intervention)

Quantitative data will be screened for missing cases, outliers, and normality of distributions using descriptive statistics. Main analyses will comprise multilevel models (i.e., MLM, mixed effects model) (39). MLM examines the effect of time (after receiving intervention) on dependent variables (changes in participants, organizational payoffs). We will include sources of random variability at the group level accounting for between-group differences and another random effects for the individual accounting for within-person differences in the repeated measures. MLM will be adjusted for demographic variables, role (i.e., nurse, physician, administrative staff) and years of experience. Analyses will be conducted using STATA. The significance level will be set at *p* = 0.05.

Qualitative data yielded from focus groups will mirror the analysis methods used in Phase 1.

#### Phase 5: Learning and Refinement

As previously mentioned, Phase 5 will take place after the first round of intervention testing and evaluation. It will aim to refine the intervention based on results yielded in the evaluation from test 1 with support of the advisory committee, thereby creating the final version of the intervention.

### Ethics and Dissemination

This research project does not involve assessment of personal and clinical data; therefore, it was seemed exempt by the Human Research Ethics Committee of Lausanne University Hospital. It will follow the ethical guidelines outlined in the Declaration of Helsinki and is based on informed consent. All participants will receive an information sheet prior to the interview; they will be informed of the nature of the study, its objectives, the procedures involved, the expected timeline, and the potential risks and benefits it may entail. We will obtain written informed consent of participants who will participate in the intervention (6-months assessment). Regarding the exploration phase, we are conducting semi-structured interviews, mostly from distance because of the COVID-19 pandemic and we obtain informed oral consent (we have audio recording of those). Consistent with the approach described above, participants receive the information sheet prior to the interview. A sign language interpreter participate in this process with D/deaf participants to ensure content understanding. Prior to beginning the interview, the research staff carefully explains all the information included in the information sheet and provides time to the participants to ask questions and make comments. No personal clinical data are being assessed, participants being asked to talk about their experience with healthcare providers and to provide insight into how developing an intervention tailored to healthcare providers to improve their communication skills.

Several steps will be taken to ensure data protection and confidentiality. First, all data collected on participants will be retained at the host institution in locked filing cabinets and on secured protected electronic files. Second, all data collected on participants will be identified through a randomly generated, unique personal identification number (PIN). A master list of PINs will be available only to research staff involved in this project.

We will protect data against disclosure in a few ways. First, published reports will be devoid of all identifying information of participants. Further, access to data will only be granted to the research staff explicitly allowed to access them by the study project leaders. Finally, only deidentified datasets will be made available for co-investigators to run potential additional secondary analyses.

## Discussion

This paper describes the protocol of an action research study aimed at developing and evaluating a capacity building intervention for healthcare staff to increase their communication skills and awareness of D/deaf and hard of hearing individuals experience and specific needs. Action research applies to this research project for several reasons. It is an appropriate method to solve practical problems; improve healthcare and professional practices; develop knowledge and new skills in healthcare and evaluate changes through cycles of practice and reflection (40).

Through its participative approach, action research empowers both communities and stakeholders. Past research highlighted the potential of this approach to address health disparities in minority groups (41), including the target population of D/deaf and hard of hearing individuals (42). Action research encourages stakeholders and communities actively participating in decision-making thorough every phase of the research project, which both empowers and supports them. Accordingly, stakeholders and D/deaf and hard of hearing individuals will be actively involved in each phase of the research project.

### Significance

In addition to experiencing significant health inequities (6–11), D/deaf and hard of hearing individuals commonly face barriers in accessing and benefiting from health care services (12, 13). Among those, barriers stemming from communication difficulties with healthcare providers are the most important (14–16). It is therefore critical to improve communication skills and awareness of D/deaf and hard of hearing individuals in healthcare providers to help decrease barriers and improve care when accessing healthcare services.

This research project will indirectly tackle access to care among D/deaf and hard of hearing populations in several ways. According to Levesque and colleagues model, healthcare access depends on five dimensions of services (i.e., *approachability; acceptability; availability and accommodation; affordability; appropriateness*) and five corresponding patients' abilities (i.e., *ability to perceive; ability to seek; ability to reach; ability to pay; ability to engage*) (43). Specific skills and knowledge developed through the intervention might affect both acceptability and ability to seek, as well as appropriateness and ability to engage. Acceptability refers to cultural and social factors making the services acceptable, whereas ability to seek healthcare includes factors determining the intention to seek care. Ability to seek health care relates to the extent to which care is organized and provided in a way that meets patients' specific needs. Accordingly, by improving awareness of D/deaf and hard of hearing experience, needs and knowledge of D/deaf culture in healthcare providers, the intervention may improve services acceptability and thereby patients' intentions to seek care.

Service appropriateness refers to the fit between patients' needs and health care provided and relates to its technical and interpersonal quality. Relatedly, ability to engage in care refers to the patients' participation, involvement and motivation, which relates to the communication capacity. Hence, by improving communication skills through the intervention, healthcare providers might provide cares that better fit patients' specific communication needs, which might in turn increase their involvement and motivation regarding treatment.

### Challenges and Limitations

The participative approach of the research project is a strength because by involving communities, stakeholders and researchers in an equitable way, it enables positive and lasting changes (41). However, this approach will bring its own challenges, such as navigating multiple relationships and handling differences in values and expectations that might arise across D/deaf, hard of hearing individuals, stakeholders and researchers. To face these challenges, regular meetings will be organized to facilitate communication. Conducting the research project will require research knowledge and experience, flexibility, pragmatism, open-mindedness, as well as strong communication and negotiation skills.

Furthermore, although past research successfully involved D/deaf and hard of hearing communities together (15, 44), this represents another potential challenge. Beyond the shared disabling loss, there are important distinctions among individuals with hearing loss stemming from used languages (i.e., spoken language with supportive tools vs. sign language). Qualitative analysis will be carried out with attention paid to the difference that will likely emerge in the data obtained from the two populations. The latter will be taken into account when developing the content and design of the intervention.

Another expected challenge pertains to the barriers that may exist among healthcare providers to attend the intervention. Healthcare providers are often overworked with limited time to attend trainings; furthermore, they may have other priorities, which could mitigate their motivation to attend the intervention. To address this challenge, we created partnerships with 5 medical institutions involved with the target population, in which we will test the intervention. If preliminary findings from this research project show that the intervention is promising, future work will be necessary to find means to further implement the intervention in ways that are compatible with healthcare providers' schedule in order to reach the most of them.

It is also important to consider the limitations of this research project. Regarding the intervention assessment, the design of this study allows to evaluate healthcare providers' trajectories after receiving the intervention. A well-known limitation related to this design is regression to the mean. We believe however that the proposed within-subjects' design is consistent with the aim to develop and provide a first evaluation of the intervention. To address this limitation, we will use qualitative data to complement quantitative findings. That said, future research conducting a randomized trial will be necessary to provide evidence for efficacy.

## Conclusion

D/deaf and hard of hearing individuals represent a vulnerable population multiply affected by medical and psychological problems. Healthcare providers often lack communication skills and knowledge related to D/deaf and hard of hearing individuals' specific needs, which relates to decreased access to healthcare (12, 15, 24, 25). In response, this research action project aims to develop and evaluate an intervention for healthcare providers aimed at increasing their awareness of D/deaf and hard of hearing individuals distinct experience and needs and to improve their communication skills with this population. The resulting intervention may contribute to decrease barriers in accessing and benefiting from care in these populations.

## Ethics Statement

This research project was deemed exempt by the Human Research Ethics Committee of Lausanne University Hospital because it does not involve clinical data measurement. It will follow the ethical guidelines outlined in the Declaration of Helsinki and is based on informed consent. Participants will receive an information sheet prior to the interview; they will be informed of the nature of the study, its objectives, the procedures involved, the expected timeline, and the potential risks and benefits it may entail.

## Author Contributions

PB and PSi conceived the study and critically reviewed the manuscript. MK helped conceive the study and its design, conducted parts of the background literature review, drafted parts of the manuscript and critically reviewed the manuscript. MG helped to assemble the measures and critically reviewed the manuscript. OC, KM, and MM conducted parts of the background literature review and critically reviewed the manuscript. PSm and TS critically reviewed the manuscript. VG helped conceive the study design, conducted parts of the background literature review, assembled measures and drafted the manuscript. All authors read and approved the manuscript.

## Conflict of Interest

The authors declare that the research was conducted in the absence of any commercial or financial relationships that could be construed as a potential conflict of interest.

## Footnotes

^1^This study is part of a larger project including two additional and non-directly related goals (1) developing a tool to identify D/deaf and hard of hearing patients within the healthcare system; (2) identify optimal approaches to disseminating information on the healthcare system and medical rights to D/deaf and hard of hearing individuals and their relatives.

## Abbreviations

IMTEE: Integrated Model of Training Evaluation and Effectiveness
MLM: Multilevel mixed effects model.
